# Renal Disease and the Heart

**DOI:** 10.14797/mdcvj.1123

**Published:** 2022-09-06

**Authors:** Hassan N. Ibrahim

**Affiliations:** 1 Division of Kidney Diseases, Hypertension & Transplantation, McGovern Medical School, University of Texas Health Science Center at Houston, Houston, Texas, US

**Keywords:** kidney, heart, venous pressure, ACE inhibition, edema

Designated as an emerging epidemic in 1997, heart failure (HF) remains a major clinical and public health problem. An estimated 64.3 million people are living with HF worldwide, and data from the National Health and Nutrition Examination Survey (NHANES) estimated HF prevalence in the United States (US) to be 2.5% based on self-reported data.^1,2^ A meta-analysis based on echocardiographic screening studies in the general population—thus also counting previously unrecognized cases—showed that the prevalence of “all type” HF in developed countries is around 11.8% in those aged 65 years and older.^3^ Notwithstanding variances in diagnostic criteria, most studies estimated that over half of all HF patients in the general population have a preserved left ventricular ejection fraction (LVEF).^4^ Data suggest that the incidence of HF is mostly flat or declining but that the burden of mortality and hospitalization remains unabated despite many significant advances.^5^

Understanding how HF leads to edema and reduction in the glomerular filtration rate has been the subject of study for decades. Perhaps one of the most elegant appraisals of this phenomenon was stated by John Peters in his 1948 landmark study: “The evidence is far from complete; but what there is points to the fact that the kidneys react to changes in the volume of the circulating blood, but are indifferent to changes in the volume of the body fluids at large except in so far as these are reflected in simultaneous disturbances of blood volume. It is not mere idle speculation to suspect that they respond only when they are apprised of a dislocation of fluid volume by its effect on the circulation in which they share…It is, therefore, logical that the kidneys should appear to be more consistently sensitive to disturbances of composition than to disturbances of volume of body fluids.”^6^

Regulatory systems, which are normally involved in homeostasis, can take on maladaptive roles. This paradoxical situation has been convincingly demonstrated in HF. The evidence is strongest with respect to the beneficial effects of sympathetic and renin-angiotensin-aldosterone system blockade. Activation of these regulators had been viewed as a compensatory response to circulatory embarrassment, but the clinical evidence proved that at some level the activation actually further impairs function. Although the success of drugs such as angiotensin-converting enzyme (ACE) inhibitors was initially attributed to hemodynamic actions unloading the left ventricle, additional direct cellular effects on cardiac remodeling have been discovered.^7^ Hyperaldosteronism regularly accompanies HF. Both increased production and decreased metabolism contribute, although increased secretion is more dominant. Aldosterone production by the heart has been reported to be elevated among patients with HF and also in rats with experimental myocardial infarction. The elevated aldosterone levels in heart failure have been convincingly implicated in progressive myocardial damage. ACE inhibition, angiotensin II blockers, and aldosterone antagonists improve the course of HF and have represented standard therapy for more than two decades. Most recently, SGLT-2 inhibitors have been shown to be remarkably efficacious in patients with heart and kidney disease regardless of left ventricular function.

In this issue, Dr. Alvaro Tamayo-Gutierrez et al. discuss the often-ignored impact of central venous congestion seen in HF patients on renal hemodynamics and highlight the pharmacology of loop diuretics, current understanding of diuretic resistance, and controversies regarding decongestive treatments. Next, Drs. Arvind Bhimaraj, Syed Adeel Ahsan, and colleagues review the expanding need and indications for combined heart-kidney transplantation and discuss appropriate indications and selection criteria, overall and organ-specific outcomes, and future perspectives.

Many patients afflicted with HF depend on left ventricular assist devices (LVAD) as a bridge to transplant or destination therapy. Drs. Ashrith Guha, Lamees El Nihum, and colleagues provide a critical appraisal of the pathogenesis of cardiorenal syndrome, the impact of preoperative renal dysfunction in patients undergoing LVAD implantation, and the effect of LVAD implantation on postoperative renal function. Then, Dr. Horacio Adrogue examines amyloidosis in the heart and kidney and the need for early diagnosis to improve long-term survival.

Almost one out of five lung and heart transplant recipients develops kidney failure in the first 10 to 15 years after transplantation. Dr. Carlos Zapata et al. review the epidemiology and causes of chronic kidney disease (CKD) after heart and lung transplantation, explore recipient demographics and comorbidities linked to CKD development, and discuss therapies and nephroprotective strategies. Hypertension has been repeatedly identified as a risk factor for kidney transplant loss and, of course, cardiovascular disease (CVD) in patients with CKD. Dr. Sean Hebert summarizes current guidelines regarding the optimal level and management of blood pressure in kidney transplant recipients.

Cardiovascular disease is the number-one cause of death in patients with CKD. Most transplant centers screen asymptomatic CKD patients presenting for kidney transplant evaluation with both invasive and noninvasive methods despite the lack of supportive evidence for such practice. Drs. Angelina Edwards and Elise Ewing comprehensively review relevant literature regarding the utility of screening for CVD in those needing a kidney transplant.

SGLT-2 inhibitors have now established themselves as superb agents for prevention of progressive kidney disease and a multitude of hard CVD end points. Drs. Abhishek Kansara, Faiza Mubeen, and Jawairia Shakil thoughtfully review the mounting evidence supporting a center-stage role for those agents beyond their effective antidiabetic capacity.

Lastly, contrast nephropathy remains a problem with no therapeutic options, although multiple preventive strategies have been tested with variable success. Focusing on prevention, authors Neil Kleiman, Isaac Tea, and Salima Gilani provide a comprehensive appraisal of the latest pre- and periprocedural strategies to minimize the risk of contrast-associated acute kidney injury in patients undergoing percutaneous coronary intervention.

It is worth reflecting on John Peters’ seminal 1948 paper in the *New England Journal of Medicine*, wherein he explains the derangements that occur in the setting of HF that lead to edema formation: “My object has been not so much to solve, as to pose, the problems of cardiac edema. I hope that I have clearly distinguished in the discussion fact from speculation. I have tried to emphasize that in the clinic, as in the laboratory, plausibility is no substitute for sound reasoning based on fundamental scientific principles; that generalization from particulars is dangerous; and that no single organ or system in a complex integrated organism can be considered in vacuo apart from the whole.”^6^ In this issue, we hope to have done the same: simply pose the issues surrounding the interaction between HF, therapies employed to alleviate its dire nature, and kidney function. We encourage you to visit journal.houstonmethodist.org to read additional content and leave comments to further this important conversation.

## Editor Biography

The editors of the *Methodist DeBakey Cardiovascular Journal* express our appreciation to Dr. Hassan N. Ibrahim for his knowledge and insight in curating this issue on kidney disease and the heart.

## Hassan N. Ibrahim, MD, MS


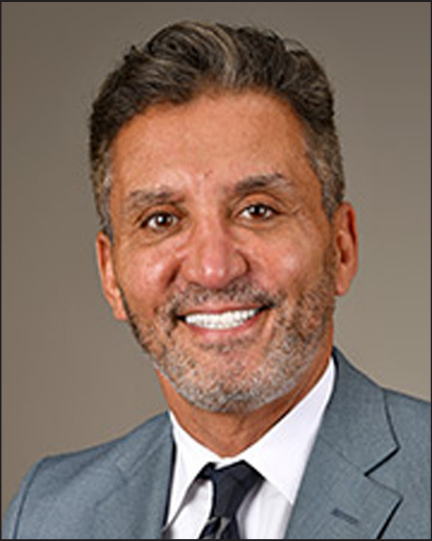


Dr. Ibrahim is a recognized national and international leader in kidney transplantation research and clinical service. Prior to joining McGovern Medical School at the University of Texas Health Science Center at Houston as a professor of surgery and director of transplant nephrology in 2022, Dr. Ibrahim was the Charles and Anne Duncan Centennial Chair in Nephrology, director of the Living Donor Kidney Transplant Program, director of the Transplant Nephrology Fellowship, and associate chairman of Academic Affairs at Houston Methodist Hospital. He also was a professor of medicine and the chief of nephrology and transplantation at the University of Minnesota.

Dr. Ibrahim’s research interests include mechanisms of kidney disease progression, kidney transplantation, and outcomes of kidney donors. His decades-long focus has helped kidney donors and clinicians determine long-term risks. He has authored more than 100 peer-reviewed papers, book chapters, and invited reviews. In addition, he has lectured on kidney transplantation and donation at numerous academic institutions and conferences worldwide.

Dr. Ibrahim has served continually on study sections for the National Institutes of Health, the Center for Disease Control, the National Kidney Foundation, and the American Society of Nephrology.
